# A modified D‐galactose‐induced aging rat model with low‐dose intraperitoneal ozone: Redox, functional, and skin outcomes

**DOI:** 10.1002/ame2.70271

**Published:** 2026-07-29

**Authors:** Isabella Kurnia Liem, Raisa Nauli, Firda Asma’ul Husna, Sasanthy Kusumaningtyas, Deswaty Furqonita, Ferbian Milas Siswanto, Soegianto Ali

**Affiliations:** ^1^ Department of Anatomy, Faculty of Medicine Universitas Indonesia Jakarta Indonesia; ^2^ School of Medicine and Health Sciences Atma Jaya Catholic University of Indonesia Jakarta Indonesia; ^3^ Department of Biochemistry, School of Medicine and Health Sciences Atma Jaya Catholic University of Indonesia Jakarta Indonesia; ^4^ Department of Biology, School of Medicine and Health Sciences Atma Jaya Catholic University of Indonesia Jakarta Indonesia

**Keywords:** D‐galactose, ozone, physical performance, redox modulation, skin outcome

## Abstract

Aging impairs quality of life, making experimental models essential to evaluate interventions that modulate age‐related changes. This study aimed to characterize a modified D‐galactose‐induced aging model and explore the effects of intermittent low‐dose ozone exposure on redox balance and functional outcomes. Twenty‐seven 12‐week‐old male Wistar rats were randomly assigned to healthy, aging (D‐galactose induction), and ozone‐treated (D‐galactose + ozone) groups. Plasma and skin MDA levels, SOD activity, and GSH levels were measured colorimetrically. Physical performance was assessed using physical health score, morbidity proportion, body weight, muscle strength (hanging test), hair regrowth, and skin structure (Hematoxylin–Eosin and Masson's trichrome staining). Compared with healthy rats, the aging group showed increased plasma MDA levels (*p* = 0.007), MDA/SOD ratios (*p* = 0.005), and MDA/GSH ratios (*p* = 0.016). In ozone‐treated rats, plasma MDA and MDA/SOD tended to decrease compared with aging rats, while GSH tended to increase. In the skin, redox markers were comparable across groups, except for a significantly lower MDA/SOD ratio in the ozone‐treated group (*p* = 0.043) compared with the aging group. D‐Galactose induction reduced physical health (*p* = 0.017), increased morbidity, and tended to reduce body‐weight gain and hanging ability. Ozone‐treated rats showed improved physical health (*p* = 0.041), lower morbidity, accelerated hair regrowth, and preserved skin structure, particularly dermal firmness and collagen thickness (*p* = 0.013). The modified D‐galactose model partially induced redox imbalance and functional deterioration, whereas intermittent low‐dose ozone exposure tended to restore redox homeostasis, preserve physical health, accelerate hair regrowth, and maintain the skin structure without inducing supraphysiological effects.

## Graphical abstract

Male Wistar rats were subjected to d‐galactose‐induced aging, with or without intermittent low‐dose intraperitoneal ozone administration, and evaluated over 8 weeks for redox status and physical performance, including physical health score, morbidity proportion, body weight, muscle strength, hair regrowth, and skin outcome. Aging was associated with redox imbalance and oxidative damage, reflected by increased MDA levels and functional decline. Low‐dose ozone treatment tended to lower oxidative stress and improve antioxidant balance, and was associated with preserved physical performance, accelerated hair regeneration, and preserved skin structure in the aging model.

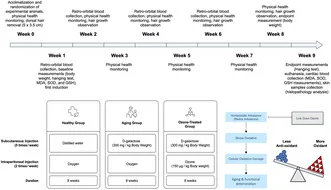

## 1 INTRODUCTION

The global increase in life expectancy and population aging has become a major public health concern due to its influence on quality of life.^1^ The World Health Organization (WHO) estimates that by 2050, the number of people ≥60 years old will reach 2.1 billion.^2^ In Indonesia, the Central Bureau of Statistics (BPS) reported in 2023 that life expectancy has risen over the past 14 years, from 67.89 years in males and 71.83 years in females in 2010 to 70.17 and 74.18 years, respectively, in 2023.^3^ Data from the Directorate of Social Welfare Statistics (2021–2023) further showed that older adults (>60 years) represent the age group with the highest prevalence of health complaints (41.49%).^4^

One causal factor that plays a central role in aging and age‐related diseases is oxidative stress. It arises when the existence of reactive oxygen species (ROS) exceeds the body's capacity to neutralize it. Accumulation of ROS will cause oxidative damage, including DNA alteration, peroxidative damage of lipids, and protein degradation that lead to cellular aging and its functional effects. Systematically, this condition can be detected by the elevation of malondialdehyde (MDA) levels and a decrease in antioxidant enzyme activity, such as superoxide dismutase (SOD), catalase (CAT), and glutathione peroxidase (GPx).^5^

Ozone has been proposed as a potential intervention to counteract aging by ameliorating oxidative stress. Medical ozone attenuates oxidative damage by regulating the H_2_O_2_ level, increasing the SOD and CAT activities, thereby reducing protein and lipid oxidation.^6^ Low to moderate doses of ozone improve mitochondrial function through activation of the NRF2/ARE signaling pathway, without causing cellular damage. Therapeutic ozone upregulates NRF2 expression, enhances antioxidant enzyme activity (SOD, CAT, GPx), and reduces mitochondrial ROS generation.^7^ This mechanism interrupts the vicious cycle linking ROS and mitochondrial dysfunction; consequently, cell survival increases. While the phenomena suggest a possible preventive role of ozone in aging‐related processes, the optimal dosing and administration interval remain incompletely understood.

D‐Galactose (D‐gal)‐induced aging rat models have been widely used due to their ability to mimic oxidative stress‐driven aging features; However, the dose and duration of induction varied, resulting in differences in the animals' physical condition and resilience to the test.^8^, ^9^ In research using the mouse as a model, a short induction time, with a dose that is acceptable to the mouse body, while still producing a representative aging model, is very important. According to the review by Sadigh‐Eteghad et al.^8^ D‐gal doses ranged from 60 to 1,250 mg/kg body weight (BW), and exposure durations ranged from 7 to 112 days, with administration via subcutaneous or intraperitoneal injection. Hadzi‐Petrushev et al.^10^ reported that antioxidant enzyme activity is affected by both D‐gal exposure and the developmental, stage of the rats. Their research results suggested using a three‐month‐old rat or older for senescence induction.

Based on the above considerations, an experimental study was developed to characterize a modified D‐galactose‐induced aging model in male Wistar rats combined with intermittent low‐dose ozone exposure. A high dose of D‐gal was administered to achieve efficient aging induction while maintaining physiological tolerance. In parallel, intermittent low‐dose intraperitoneal ozone exposure was introduced as an exploratory redox‐modulating intervention. Redox biomarkers and functional outcomes were subsequently assessed to evaluate the biological relevance of the model and the effect of ozone exposure.

## 2 METHODS

### 2.1 Animals

Twenty‐seven healthy, 12‐week‐old male Wistar rats were obtained from PT Biofarma (Bandung, West Java, Indonesia). Animals were housed under controlled environmental conditions (21°C; 12‐h light/dark cycle) with free access to food and water. All experimental procedures were conducted in accordance with animal welfare guidelines, and animal well‐being was carefully monitored throughout the study. The number of research subjects was determined using the resource equation approach formula plus a 10% dropout (because of death) probability.

### 2.2 Experimental procedure

The total experimental duration was 10 weeks, including 1 week for acclimatization, 8 weeks for intervention, and 1 week for post‐intervention. The rats were randomly assigned to three groups (*n* = 9 per group): healthy control, aging, and ozone‐treated. Following acclimatization, a 5 cm × 3.5 cm dorsal area was shaved to assess hair regrowth. Aging was induced in the aging and ozone‐treated groups by daily subcutaneous injections of 300 mg/kg body weight (bw) D‐gal (Sigma‐Aldrich, 30750, MO, USA) 5 days/week for 8 weeks. The healthy control group received an equivalent volume (1 mL) of distilled water. Concurrently, the ozone‐treated group also received an intraperitoneal injection of 150 μg/kg bw ozone (O_2_/O_3_) twice weekly for 8 weeks, whereas the healthy and aging groups received an intraperitoneal injection of oxygen (2 mL) at the same frequency. Observation of physical condition and body‐weight measurements were undertaken every day (5 days/week). The hanging test was evaluated before (week 0) and after the intervention (week 9).

Heparinized blood samples were collected under general anesthesia by an intraperitoneal injection of ketamine (60 mg/kg BW) and xylazine (5 mg/kg BW). Blood samples were obtained via the retro‐orbital plexus at weeks 0, 2, 4, and 6. At the end of the experiment (week 9), terminal blood collection was performed by cardiac puncture under general anesthesia. Subsequently, dorsal skin samples were collected from the shaved area and divided into two parts. One part was stored at −20°C for biochemical analysis, while the other part was fixed in 10% Neutral Buffer Formalin (NBF) for histopathological evaluation.

### 2.3 Plasma and skin homogenate preparation

Plasma was separated from heparinized blood by centrifugation at 1500 × *g* for 10 min at room temperature. For preparation of 10% (w/v) skin homogenate, tissue samples were homogenized in ice‐cold 0.01 M Phosphate Buffer Saline (PBS) pH = 7.4 (Thermo, P4417‐50TAB, MA, USA) followed by centrifugation at 5000 × *g* for 5 min at 4°C. The resulting supernatant, together with plasma samples, was used for MDA, GSH, and SOD activity quantification.

### 2.4 Measurement of oxidative stress and antioxidant biomarkers

Oxidative stress and antioxidant biomarkers, including MDA, reduced glutathione (GSH), and SOD activity, were assessed using a colorimetric method. MDA levels were quantified by reacting plasma samples with thiobarbituric acid (TBA) to produce thiobarbituric acid reactive substances (TBARS), with absorbance measured at 530 nm. Meanwhile, GSH levels were quantified using Ellman's method based on the reaction between GSH and 5,5′‐dithiobis‐(2)‐nitrobenzoic acid (DTNB) (Thermo, 22582, IL, USA), which produces a yellow‐colored solution, with absorbance being measured at 420 nm. All MDA assay solutions were provided in‐house by the Department of Biochemistry and Molecular Biology, Faculty of Medicine, Universitas Indonesia. SOD activity was measured using the total superoxide dismutase (T‐SOD) activity assay kit (Elabscience, E‐BC‐K019‐S, TX, USA) following the manufacturer's protocol. For skin homogenate samples, MDA, GSH level, and SOD activity were normalized to total protein content determined by the Bradford assay (Biorad, 5000006, CA, USA).

### 2.5 Physical performance test

Physical performance was evaluated based on physical health, morbidity, body‐weight gain, muscle strength, and hair regrowth. The subject's physical health was defined as the percentage of the observation period (10 weeks; weeks 0–9) during which the animal remained in good physical condition without symptoms or signs of illness divided by the total duration of observations (10 weeks, week 0 to 9), expressed as a percentage. The morbidity rate was defined as the total number of subjects with symptoms or signs of illness divided by the total number of subjects per group. Body weight was evaluated at 2‐week intervals throughout the study. Muscle strength was assessed using a hanging test on a custom apparatus (25 cm × 25 cm × 50 cm). The latency to fall was recorded across three attempts per animal, and the average value was used for analysis. The hair regrowth was visually monitored and documented every 2 weeks under general anesthesia. The observed hair growth rate was measured semi‐quantitatively using the following criteria: 0: no growth, 1: growth <25% area, 2: growth 25%–50% area, 3: growth 50%–75% area, and 4: growth 75%–100% area.

### 2.6 Skin histopathological evaluation

The shaved skin samples taken from the back (approximately 1 cm^2^) were immediately fixed in 10% neutral buffer formalin (NBF) at room temperature for approximately 24 h, followed by routine tissue processing through graded alcohol dehydration, xylene clearing, paraffin infiltration, and embedding. The paraffin blocks were sectioned at 4 μm thickness using a microtome (Leica, Germany). Tissue sections were then stained with hematoxylin and eosin (H&E) to evaluate the epidermal thickness, dermal thickness, fibroblast cell number, and hair follicle number, and with Masson's trichrome (MT) to assess dermal collagen thickness. All the histopathological preparation and staining were done at the Department of Pathological Anatomy, Faculty of Medicine, Universitas Indonesia (Jakarta, Indonesia). All slides were examined under a light microscope (Olympus BX53, Tokyo, Japan). Photomicrographs were prepared using an Olympus camera (Olympus, Tokyo, Japan).

In H&E staining, the cytoplasm is tipically colored pink, and the nucleus is colored blue. In Masson's trichrome staining, collagen fibers appeared blue or green, and cytoplasm and muscle fibers appeared pink. Epidermal and dermal thicknesses were measured at 40× and 100× magnification, respectively, at five randomly selected points within a single field of view. Fibroblasts were counted in five fields of view at 400× magnification within an area of 75 000 μm^2^, while hair follicles were counted in five fields of view at 100× magnification within an area of 1.22 mm^2^. Dermal collagen thickness was measured on MT‐stained sections at 40× magnification at five points within a single field of view. All morphometric analyses were performed using Olympus Stream software version 1.6. Histopathological evaluation was done by an expert from Balivet Laboratory (Bogor, Indonesia).

### 2.7 Statistical analysis

Normally distributed data were analyzed using one‐way ANOVA and Tukey's HSD post‐hoc tests, whereas non‐normally distributed data were analyzed using the Kruskal‐Wallis test followed by Dunn's post‐hoc test. Statistical significance was set at *α* = 0.05. Proportion data were analyzed using Fisher's exact test (2 × 2 contingency table) with a 95% confidence interval (Cl) and relative risk (RR) calculation. Hair regrowth was reported descriptively. Spearman's rank correlation analysis was performed to evaluate the relationship between systemic redox status and physical health condition. All measurements, including molecular markers (MDA, SOD, GSH), physical performance tests, and skin histopathology analyses, were conducted on the 19 surviving rats. The data analyses were performed using GraphPad Prism software (version 10.1).

## 3 RESULTS

### 3.1 Redox status shift in the modified D‐galactose aging induction and ozone intervention

**FIGURE 1:**
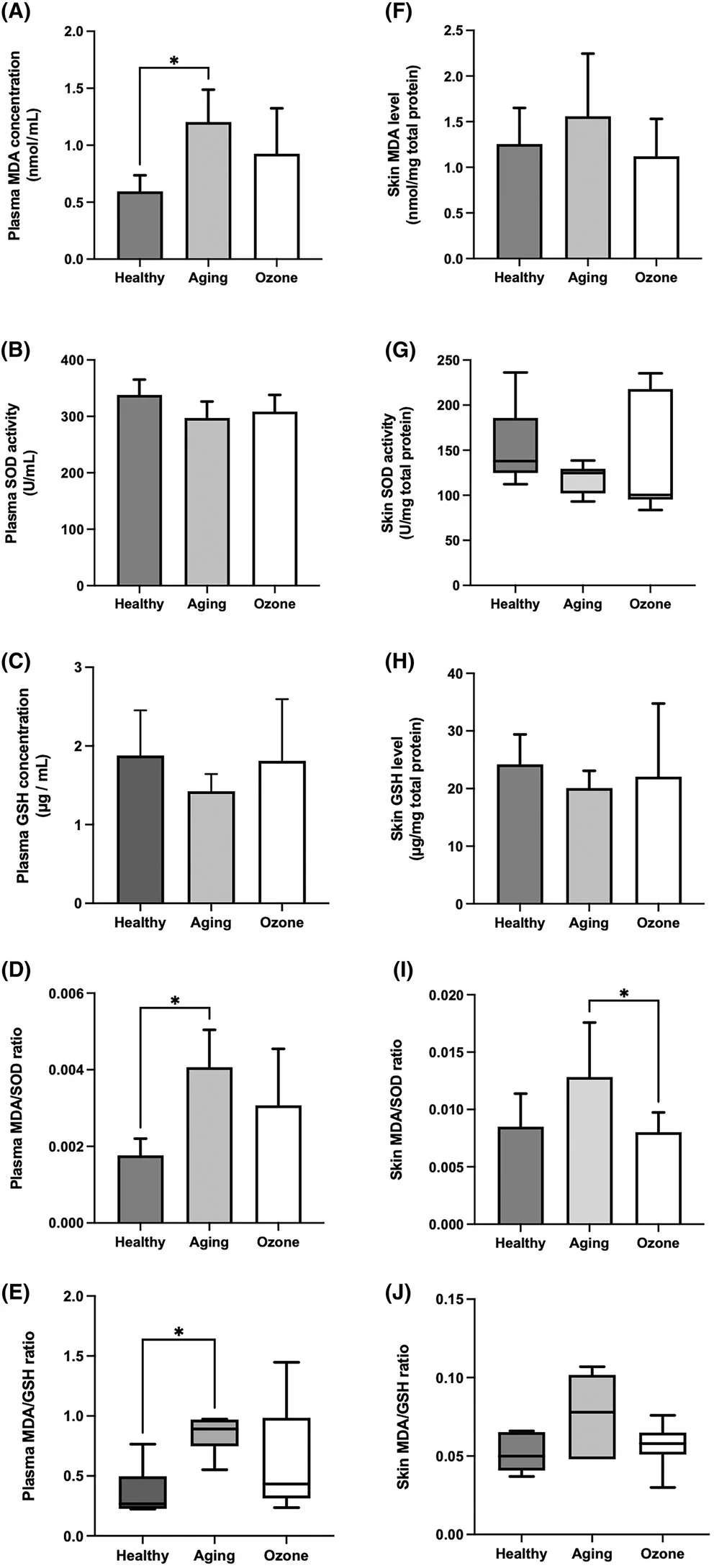
Systemic and cutaneous redox balance status. Plasma concentrations of (A) MDA; (B) SOD activity; (C) GSH; (D) MDA/SOD ratio; and (E) MDA/GSH ratio. Skin levels of (F) MDA; (G) SOD activity; (H) GSH; (I) MDA/SOD ratio, and (J) MDA/GSH ratio. Data are presented as mean ± SD (One‐way ANOVA followed by Tukey’s test; *p < 0.05) or box and whisker plots (min to max; Q1 to Q3) (Kruskal‐Wallis with Dunn’s test; *p < 0.05).

The plasma MDA concentration in the aging group was significantly higher than the healthy group (*p* = 0.007; Figure 1A). Plasma SOD activity (*p* = 0.063; Figure 1B) and GSH (*p* = 0.396; Figure 1C) were lower than the healthy group, but not statistically significant. Both the plasma MDA/SOD (*p* = 0.005; Figure 1D) and MDA/GSH ratios (*p* = 0.016; Figure 1E) were elevated in the aging group, with a significant increase observed compared to the healthy group. Ozone treatment tended to improve systemic redox status. The ozone‐treated group showed a tendency toward lower MDA levels (*p* = 0.251), with higher SOD activity (*p* = 0.766), higher GSH concentrations (*p* = 0.482), lower MDA/SOD ratios (*p* = 0.253), and lower MDA/GSH ratios (*p* = 0.588) than the aging group. However, those changes were not statistically significant.

A similar pattern was observed in the skin tissue analysis (Figure 1F–J). Compared with the healthy group, the aging group showed higher MDA levels (*p* = 0.568; Figure 1F) with lower SOD activity (*p* = 0.371; Figure 1G) and lower GSH concentrations (*p* = 0.684; Figure 1H). Skin tissue from the aging group also tended to show higher MDA/SOD ratios (*p* = 0.086, Figure 1I) and MDA/GSH ratios (*p* = 0.472; Figure 1J) compared to the healthy group. Ozone treatment tended to reduce MDA levels (*p* = 0.298), increase SOD activity (*p* = 0.999), and GSH (*p* = 0.910) concentrations relative to the aging group. Notably, ozone treatment significantly decreased the skin MDA/SOD ratio (*p* = 0.043) compared with the aging group. The MDA/GSH ratio also showed a decreasing trend compared to the aging group (*p* = 0.999), although the changes were not statistically significant.

### 3.2 Changes of physical performance in aging and ozone‐treated groups

**FIGURE 2:**
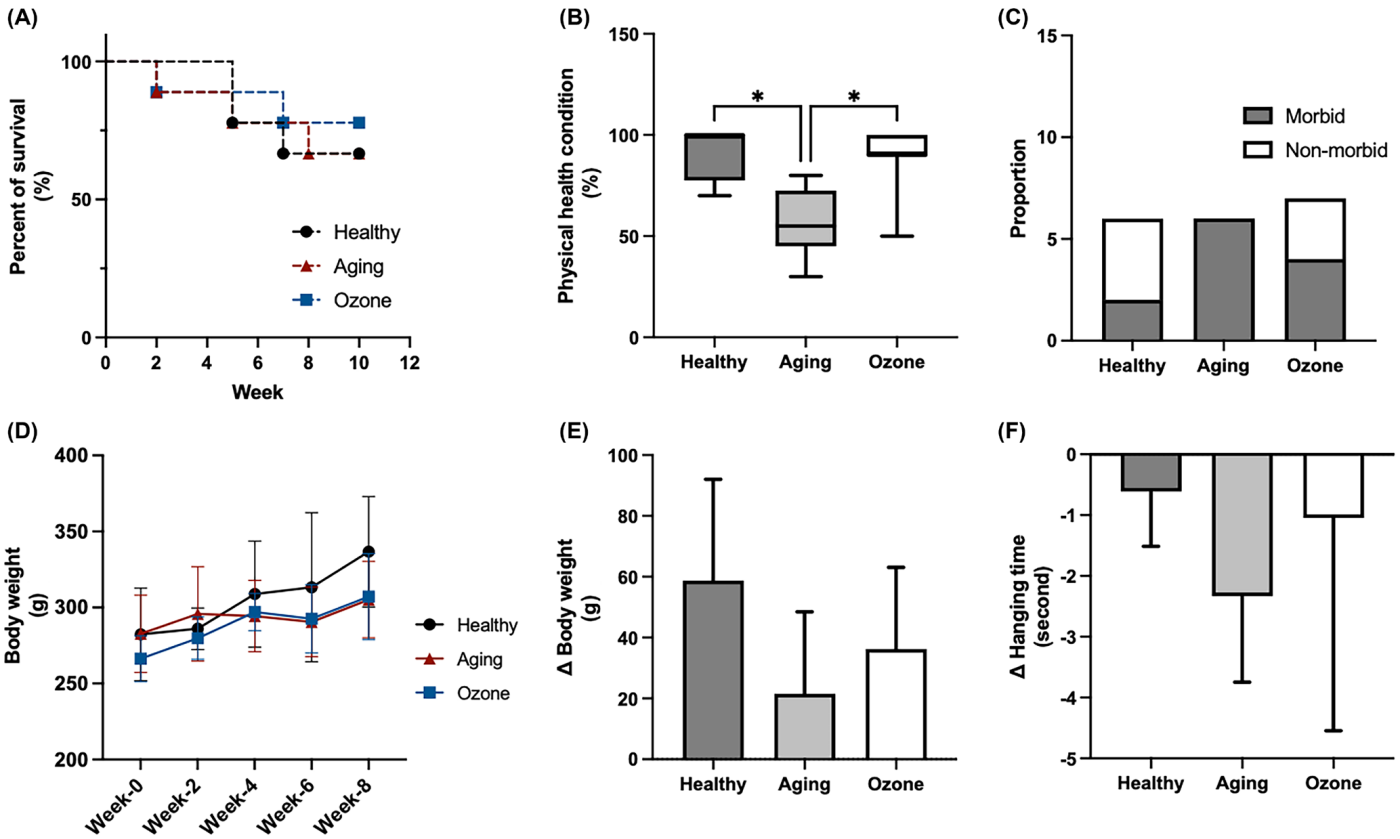
Physical performance analysis. (A) Percentage survival over a 10‐week experimental period. (B) Physical health condition. Data are presented as median, min to max (Kruskal‐Wallis, Dunn's test, *p < 0.05). (C) Morbidity proportion (Fisher's exact test). (D) Body weight changes. (E) Delta body weight (ΔBW). (F) Delta hanging time. Data are presented as mean ± SD. ΔBW and hanging times were calculated as the differences between the values at weeks 8 and 9 and the baseline (week 0) values.

Deaths occurred in each group over the study period. A total of 8 rats (healthy control: 3, aging: 3, ozone‐treated: 2) were lost because of death, giving a final total of 19 rats (*n* = 6 for healthy and aging groups, *n* = 7 for ozone‐treated group). Deaths occurred in the 2nd (*n* = 2), 5th (*n* = 3), 7th (*n* = 2), and 8th week (*n* = 1) (Figure 2A). Most deaths were preceded by the onset of symptoms, namely sneezing, shivering, coughing, and/or weakness and inactivity. Only 2 rats died suddenly, in the 5th week without any observed symptoms or signs of illness.

Physical health conditions significantly differed among groups (Kruskal‐Wallis, *p* = 0.005, Figure 2B). The median percentage of physical health of the aging group significantly decreased (*p* = 0.017) to 55 (min–max 30–80) compared to the healthy control (100; min–max 70–100). The ozone‐treated group exhibited a significantly higher score (90; min–max 50–100) than the aging group (*p* = 0.041) and were comparable to healthy controls (*p* > 0.999). The morbidity proportion did not differ significantly among groups (*p* > 0.05; Fisher's exact test) (Figure 2C). Nevertheless, the relative risk analysis indicated a lower morbidity risk in the healthy control compared with the aging group (RR = 0.33; 95% CI: 0.10–0.76; *p* = 0.061) and a higher morbidity risk in the aging group compared with the ozone‐treated group (RR = 1.75; 95% CI: 0.94–2.19, *p* = 0.192). Morbidity risk between the healthy and ozone‐treated groups was not different (RR = 0.58; 95% CI: 0.15–1.90; *p* = 0.592). All groups gained body weight throughout the study; however, the extent of weight gain differed among the groups (Figure 2D). The aging group showed the smallest increase, followed by the ozone‐treated and healthy groups (Figure 2E). Muscle strength, assessed by hanging time, tended to decline in all groups over the experimental period (Figure 2F). The hanging time in the aging group tended to decrease compared to the healthy group (*p* = 0.428). Meanwhile, the ozone‐treated group showed longer hanging times than the aging group, although the difference was not significant (*p* = 0.594).

Spearman's rank correlation analysis showed a significant negative correlation between plasma MDA concentration and physical health condition (*r* = −0.583; *p* = 0.009). Physical health was also negatively correlated with the MDA/SOD ratio (*r* = −0.528; *p* = 0.020) and the MDA/GSH ratio (*r* = −0.584; *p* = 0.009). In contrast, SOD activity showed a weak positive correlation with physical health (*r* = 0.286; *p* = 0.234), whereas the GSH levels exhibited a moderate positive correlation (*r* = 0.497; *p* = 0.030). These findings suggest that shifts in systemic redox status may be associated with physical health, even when changes in individual biomarkers are not statistically significant at the group level.

### 3.3 Hair growth dynamics

**FIGURE 3:**
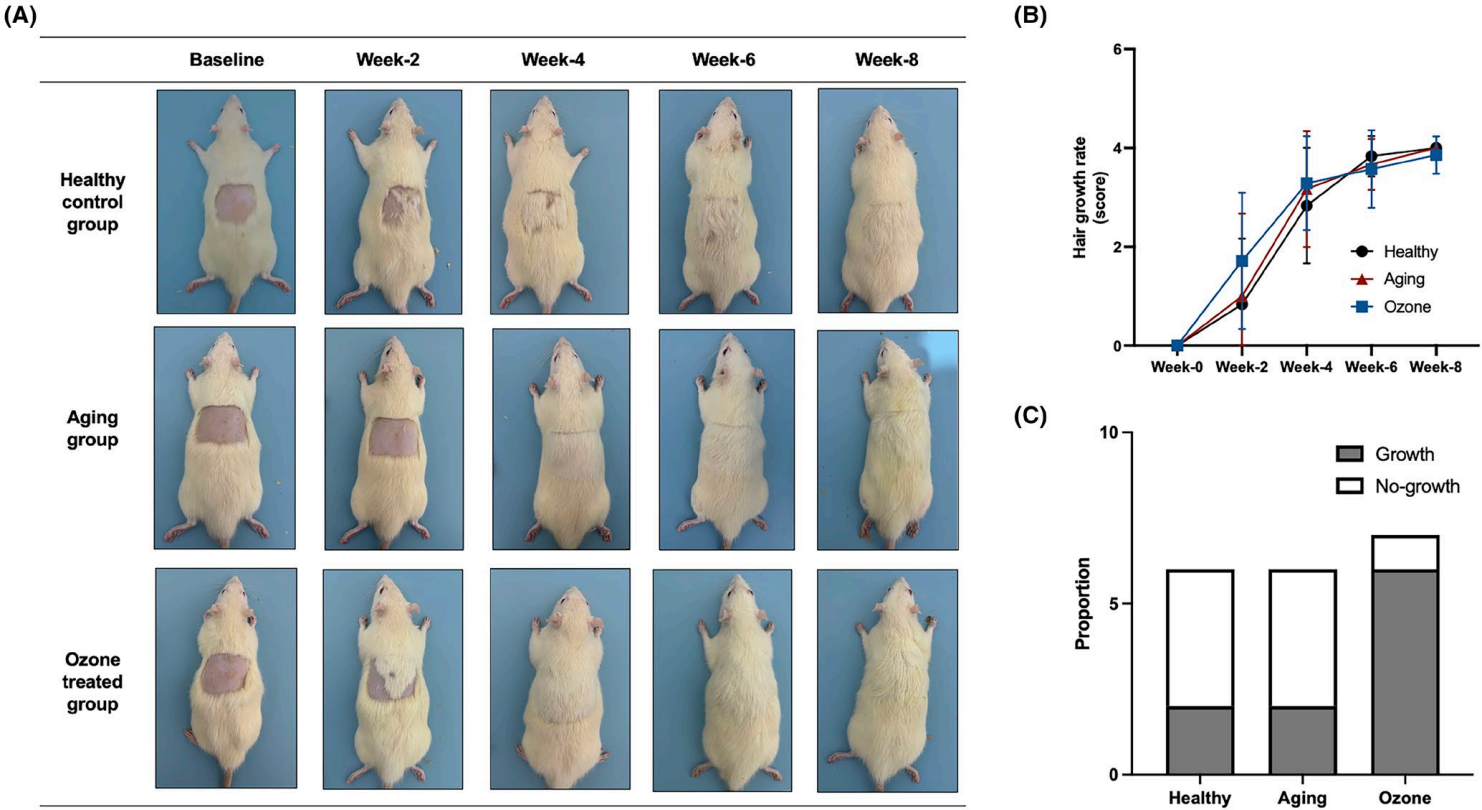
The effect of ozone treatment on hair growth in D‐galactose‐induced aging rats. (A) Visualization of hair growth from week 0 to week 8. (B) Hair growth rate scores from week 0 to week 8. (C) Proportion of animals with hair growth at week 2.

The hair of all groups was fully grown at the end of observations (Figure 3A). The growth rate score of the aging group was comparable to that of the healthy group. However, the ozone‐treated group showed a faster growth rate in week 2 (Figure 3B). In addition, the ozone‐treated group contained the highest proportion of animals with hair growth (6 from 7) compared to the other groups (2 from 6 rats; Figure 3C) at week‐2.

### 3.4 Changes in skin histopathology

**FIGURE 4:**
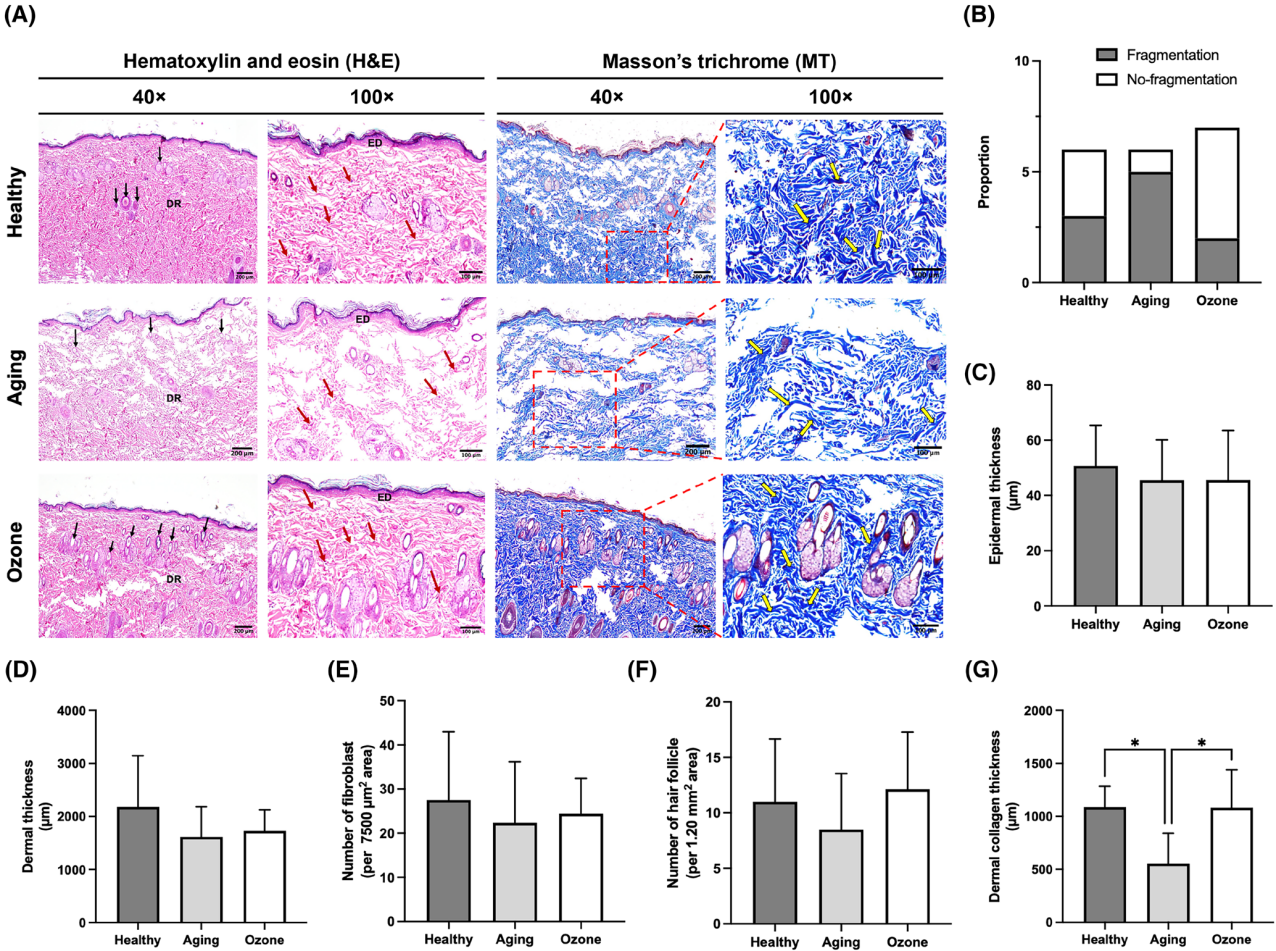
Effects of ozone treatment on skin histopathology in D‐galactose aging rats. (A) Representative H&E‐ and MT‐stained skin sections (magnifications: 40× and 100×). (B) Proportion of animals exhibiting dermal fragmentation and non‐fragmentation. Quantitative analysis of epidermal thickness (C), dermal thickness (D), fibroblast cells number (E), hair follicles number (F), and dermal collagen thickness (G) (ANOVA, Tukey's test, *p < 0.05). Data were presented as mean ± SD. DR, dermis; ED, epidermis. Black arrows indicate hair follicles; Red arrows indicate fibroblast cells; Yellow arrows indicate hyalinization.

Histological evaluation of H&E‐ and MT‐stained skin sections showed a better preservation of skin structure in the healthy and ozone‐treated groups than in the aging group (Figure 4A). Skin tissues from the aging group tend to exhibit dermal fragmentation in 83.3% (5 from 6) samples compared to the healthy (3 from 6 or 50%) and ozone‐treated (2 from 7 or 28.6%) groups (Figure 4B), suggesting extracellular matrix degradation and reduced skin integrity. Quantitative analyses showed that the aging group tended to have lower epidermal thickness (*p* = 0.839), dermal thickness (*p* = 0.335), fibroblast number (*p* = 0.761), and hair follicle number (*p* = 0.696) than the healthy group, although these differences were not statistically significant (Figure 4C–F). Fibroblasts in the aging group also exhibited a shortened morphology, and hair containing follicles were rarely observed (Figure 4A). The ozone‐treated group showed better preserved dermal structure, with elongated spindle‐shaped fibroblasts and several hair‐containing follicles (Figure 4A). While epidermal thickness (*p* = 0.999), dermal thickness (*p* = 0.952), fibroblast number (*p* = 0.953), and hair follicle number (*p* = 0.448) tended to be higher in the ozone‐treated group than in the aging group, these differences were not statistically significant (Figure 4C–F). Notably, dermal collagen thickness was significantly higher in the ozone‐treated group than in the aging group (*p* = 0.013) and was comparable to the healthy group (*p* = 0.999) (Figure 4G).

## 4 DISCUSSION

This work focused on the creation of a modified D‐gal‐induced aging rat model combined with a low dose ozone intervention to modify the redox balance and its influence on physiological outcomes. The main results are: (1) the modified D‐gal protocol could induce a redox imbalance, (2) the intermittent low‐dose ozone exposure was sufficient to modulate redox biomarkers toward a less oxidative profile, (3) the redox changes corresponded to the physical performance, onset of hair growth, and preserved skin structure with no evidence of an excessive physiological burden. Although 19 animals completed the intervention study, mortality across groups during the study indicates that the tolerability of the protocol should be interpreted cautiously.

Mortality occurred in all groups, with a comparable number of deaths. Although no clear evidence of laboratory contamination or animal husbandry failure was identified, the exact causes of death could not be determined because necropsy, microbiological testing, and histopathological examination were not performed. Therefore, undetected subclinical illness, baseline health vulnerability, or batch‐related conditions cannot be excluded. This may have weakened the validity of the healthy control baseline and confounded the interpretation of both D‐galactose‐induced aging and ozone‐associated effects. However, morbidity and health score data still showed a biologically plausible pattern, with the aging group having the highest morbidity proportion and lowest median health score, whereas the ozone‐treated group showed relatively preserved health status. Thus, the present findings should be interpreted as preliminary and require confirmation in larger studies with stricter baseline screening, systematic mortality assessment, and longitudinal monitoring.

Our modified D‐gal administration triggered a redox imbalance. The plasma MDA concentration of the aging group increased significantly compared to that of the healthy group. This implied increased lipid peroxidation as a consequence of oxidative stress, a consistent feature of the D‐gal model across organs.^10^ Plasma SOD activity tended to decrease, as did GSH; as a consequence, the MDA/SOD ratios and the MDA/GSH ratios significantly increased. As reported by Hadzi‐Petrushev et al.,^10^ the nonsignificant decrease in SOD activity still makes biological sense. The SOD responses in the D‐gal model can vary, depending on animal age, organ measured, duration/pattern of induction, and silencing of other antioxidant enzymes. Therefore, alterations in MDA levels and their ratios often serve as more sensitive indicators of redox status shifts than SOD activity alone. D‐gal induction may reduce GSH levels mainly through chronic oxidative stress. Repeated D‐gal exposure increases ROS, advanced glycation end product (AGE) formation, mitochondrial dysfunction, and inflammation, leading to greater GSH consumption for detoxifying H_2_O_2_ and lipid peroxides via GPx. During this process, GSH is oxidized to glutathione disulphide (GSSG); when ROS production exceeds GSH regeneration capacity, intracellular GSH levels decline.^10^

In addition to its systemic effects, D‐gal induction may also influence the local tissue redox status. Although the changes in skin redox markers were not statistically significant, the overall pattern suggests that D‐gal induction may shift local redox homeostasis toward a more pro‐oxidative state. The difference between plasma and skin findings may reflect the distinct nature of systemic and tissue‐specific oxidative stress assessments, as plasma MDA represents the cumulative oxidative burden across multiple organs and tissues, whereas skin MDA reflects localized redox alterations within a single tissue compartment.

Low‐dose intermittent ozone tends to modulate redox imbalance. The ozone‐treated group showed a tendency toward a decrease in plasma and skin MDA, MDA/SOD ratio, and MDA/GSH compared to the aging group. However, these reductions did not reach statistical significance. Similarly, dose‐based analysis showed that ozone administration did not significantly reduce MDA levels at any dose.^11^ Furthermore, no significant effect on MDA production was observed in subgroups based on administration method or additional treatment.^11^ Despite the lack of statistical significance, the observed trend toward lower MDA levels may indicate a reduction in lipid and oxidative damage.

The observed reduction in MDA/SOD and MDA/GSH ratios suggests a tendency toward improved oxidative‐antioxidative balance, even in the absence of significant changes in individual biomarkers. This finding was consistent with the concept of low‐dose ozone as a redox bioregulator rather than a direct antioxidant or supraphysiological stimulator.^7^ The antioxidant response is not always manifested as a marked increase in SOD or GSH alone. Rather, it may involve multiple pathways, including the glutathione system, GPx, glutathione S‐transferase (GST), heme‐oxygenase‐1 (HO‐1), or broader improvements in redox buffering capacity that are not captured by a single biomarker measurement. An animal study on intraperitoneal ozone administration suggested activation of the Nrf2 pathway and increased expression of antioxidant defense components, such as GST in certain tissues, indicating a possible mechanism underlying the antioxidant effects, although antioxidant responses may differ among experimental models and organs.^7^, ^12^

Low‐dose ozone has been proposed to induce a mild oxidative stimulus that can activate the Keap1‐like ECH‐associated protein (Keap1)‐Nrf2/ARE signaling pathway.^13^ Oxidative modification of Keap1 may facilitate Nrf2 dissociation and nuclear translocation. Leading to the transcription of multiple cytoprotective genes involved in antioxidant defense and cellular stress adaptation. This response may enhance the activity of various antioxidants, including the glutathione‐related pathway, thereby improving redox homeostasis and limiting oxidative damage.^13^, ^14^ Such a mechanism may partly explain the observed tendency toward reduced lipid peroxidation and lower oxidative‐antioxidative ratios, despite the absence of significant changes in individual parameters in the present study. The ability of ozone to restore cellular redox balance suggests a potential approach in preventing and managing age‐related diseases.^15^

The redox change might be associated with physical condition. The aging group exhibited a significant reduction in the physical health of the animals. Although the morbidity did not differ significantly among groups, the morbidity risk was consistent with the physical health outcome, with higher vulnerability observed in aging rats and a lower risk trend in ozone‐treated rats. Nevertheless, ozone exposure significantly improved the physical health score and restored values to levels comparable to those of healthy rats. These findings suggest that ozone improves functional resilience, which may precede detectable changes in overt morbidity, particularly in small experimental cohorts. In line with this, the aging group had the lowest body‐weight gain and the greatest decrease in hanging time. However, since these changes were not statistically significant, they can be assumed to be within acceptable physiological tolerance. On the other hand, ozone exposures tended to maintain body weight better than the aging group. These study results align with a study by Gama et al.,^16^ which demonstrated that mice receiving chronic D‐galactose exhibited decreased body weight, whilst Li et al.^17^ reported improved body condition and increased body weight with ozone administration.

Hair regrowth and skin structure preservation may be peripheral indicators of aging sensitive to changes in the microenvironment. Ozone exposure could promote hair growth, with a faster onset observed at week 2 post‐injection. Hair growth in the aging group was comparable to that in the healthy group but slower than in the ozone‐treated group. Histopathological observation supports this condition. Hair follicles tended to decrease in the aging group, but increased in the ozone group. The skin structures, especially the dermal resilience and collagen thickness, were significantly decreased in the aging group compared with the healthy group. Low‐dose ozone exposure appears to improve this aging skin condition, with increased dermal collagen thickness and increased dermal firmness. This result implied that our D‐gal‐induced aging model disrupts the microenvironment under acceptable physiological conditions. This finding also suggests that intermittent low‐dose ozone may prevent degenerative processes, potentially through improvements in the redox microenvironment, tissue perfusion, or inflammatory balance.^18^

Although the present findings tend to demonstrate the beneficial effects of ozone treatment on redox balance, physical performance, hair regrowth, and preserved skin structure, several limitations should be addressed. The molecular mechanisms underlying these effects were not directly examined, as key antioxidant and inflammatory mediators such as Nrf2, HO‐1, CAT, GPX, interleukin‐6 (IL‐6), and tumor necrosis factor‐α (TNF‐α) were not assessed. Future studies incorporating gene and protein expression analyses are warranted to provide a more comprehensive understanding of the molecular events involved in ozone‐mediated protection against aging‐related alterations.

## 5 CONCLUSIONS

Our modified D‐galactose protocols could induce redox imbalance, as evidenced by increased MDA concentrations, and MDA/SOD and MDA/GSH ratios. The intermittent low‐dose intraperitoneal ozone injections partially modulated cellular redox balance twardo a less oxidative profile. These changes in redox balance appear to be associated with physical performance, faster hair regeneration, and maintaining skin resilience. Nevertheless, the molecular mechanisms remain to be elucidated. This study may serve as a potential model for future anti‐aging intervention studies.

## AUTHOR CONTRIBUTIONS

**Isabella Kurnia Liem:** Conceptualization; data curation; formal analysis; funding acquisition; investigation; methodology; project administration; resources; supervision; validation; visualization; writing – original draft; writing – review and editing. **Raisa Nauli:** Data curation; formal analysis; investigation; project administration; validation; visualization; writing – original draft; writing – review and editing. **Firda Asma’ul Husna:** Investigation; project administration; writing – review and editing. **Sasanthy Kusumaningtyas:** Investigation; project administration; supervision. **Deswaty Furqonita:** Investigation; project administration; supervision. **Ferbian Milas Siswanto:** Conceptualization; methodology; supervision; validation. **Soegianto Ali:** Conceptualization; methodology; supervision; validation.

## FUNDING INFORMATION

This work was supported by a Regular Fundamental Research grant from the Ministry of Higher Education, Science, and Technology of Republic Indonesia (Grant number PKS‐484/UN2.RST/HKP.05.00/2025).

## CONFLICT OF INTEREST STATEMENT

The authors declare no conflict of interests.

## ETHICS STATEMENT

This study was approved by the Ethics Committee of the Faculty of Medicine, Universitas Indonesia (No. KET‐452/UN2.F1/ETIK/PPM.00.02/2025) and the Ethics Committee of the Faculty of Medicine and Health Sciences, Atma Jaya Catholic University of Indonesia (No. 18/05/KEP‐FKIKUAJ/2025).

## Data Availability

Research data are not shared.
